# Reappraisal of intra-abdominal candidiasis: insights from peritoneal fluid analysis

**DOI:** 10.1186/s40635-023-00552-0

**Published:** 2023-09-30

**Authors:** Emmanuel Novy, Mathieu Esposito, Julien Birckener, Adeline Germain, Marie-Reine Losser, Marie-Claire Machouart, Philippe Guerci

**Affiliations:** 1 Service d’anesthésie-Réanimation et Médecine Péri-Opératoire, CHRU Nancy, Hôpitaux de Brabois, 54500 Vandœuvre-Lès-Nancy, France; 2 SIMPA, UR7300, Université de Lorraine, 54500 Vandœuvre-Lès-Nancy, France; 3 Service de Chirurgie Digestive, CHRU Nancy, Hôpitaux de Brabois, 54500 Vandœuvre-Lès-Nancy, France; 4 NGERE, U1256, Université de Lorraine, 54500 Vandœuvre-Lès-Nancy, France; 5 DCAC, INSERM 1116, Université de Lorraine, 54500 Vandœuvre-Lès-Nancy, France; 6 Service de Mycologie et Parasitologie, CHRU Nancy, Hôpitaux de Brabois, 54500 Vandœuvre-Lès-Nancy, France

**Keywords:** Secondary peritonitis, Candida, Virulence, Critically ill patient, Pathogenicity

## Abstract

**Background:**

The understanding of high mortality associated with intra-abdominal candidiasis (IAC) remains limited. While *Candida* is considered a harmless colonizer in the digestive tract, its role as a true pathogen in IAC is still debated. Evidence regarding *Candida* virulence in the human peritoneal fluid are lacking. We hypothesized that during IAC, *Candida albicans* develops virulence factors to survive to new environmental conditions. The objective of this observational exploratory monocentric study is to investigate the influence of peritoneal fluid (PF) on the expression of *C. albicans* virulence using a multimodal approach.

**Materials and methods:**

A standardized inoculum of a *C. albicans* (3.10^6^ UFC/mL) reference strain (SC5314) was introduced in vitro into various PF samples obtained from critically ill patients with intra-abdominal infection. Ascitic fluids (AFs) and Sabouraud medium (SBD) were used as control groups. Optical microscopy and conventional culture techniques were employed to assess the morphological changes and growth of *C. albicans*. Reverse transcriptase qPCR was utilized to quantify the expression levels of five virulence genes. The metabolic production of *C. albicans* was measured using the calScreener™ technology.

**Results:**

A total of 26 PF samples from patients with secondary peritonitis were included in the study. Critically ill patients were mostly male (73%) with a median age of 58 years admitted for urgent surgery (78%). Peritonitis was mostly hospital-acquired (81%), including 13 post-operative peritonitis (50%). The infected PF samples predominantly exhibited polymicrobial composition. The findings revealed substantial variability in *C. albicans* growth and morphological changes in the PF compared to ascitic fluid. Virulence gene expression and metabolic production were dependent on the specific PF sample and the presence of bacterial coinfection.

**Conclusions:**

This study provides evidence of *C. albicans* virulence expression in the peritoneal fluid. The observed variability in virulence expression suggests that it is influenced by the composition of PF and the presence of bacterial coinfection. These findings contribute to a better understanding of the complex dynamics of intra-abdominal candidiasis and advocate for personalized approach for IAC patients.

*Trial registration* https://clinicaltrials.gov/ (NCT05264571; February 22, 2022)

**Graphical Abstract:**

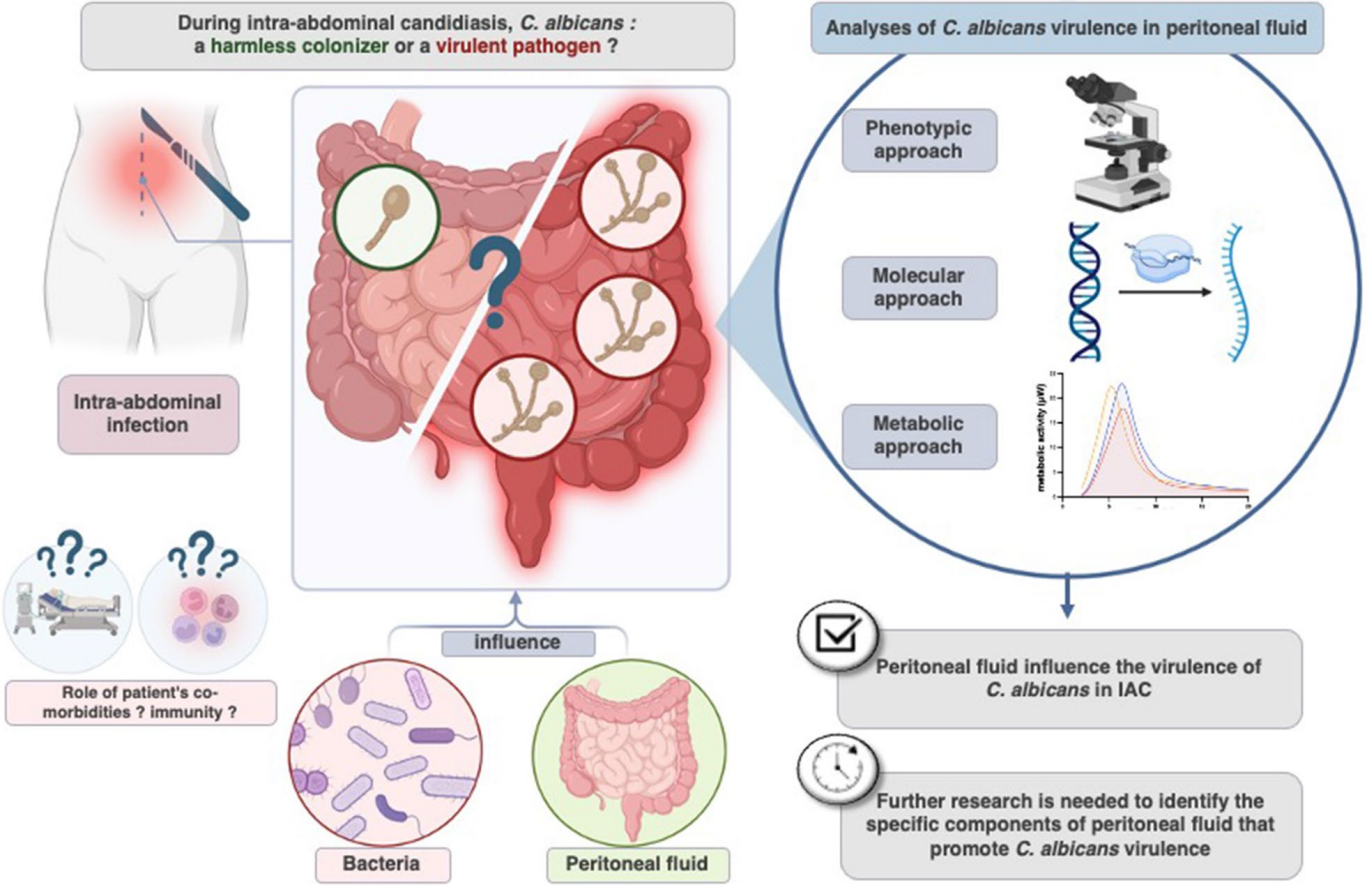

**Supplementary Information:**

The online version contains supplementary material available at 10.1186/s40635-023-00552-0.

## Highlights

- This study is the first to demonstrate the expression of *Candida albicans* virulence in human peritoneal fluid.
- The expression of *Candida albicans* virulence in peritoneal fluid shows significant variability among critically ill patients with intra-abdominal infection.
- The composition of peritoneal fluid and bacterial coinfection may influence the expression of *Candida albicans* virulence.
- These findings emphasize the imperative to reevaluate the delineation of intra-abdominal candidiasis, with due consideration to the virulence expression exhibited by isolated *Candida*.

**Supplementary Information:**

The online version contains supplementary material available at 10.1186/s40635-023-00552-0.

## Background

Intra-abdominal infections are the second cause of sepsis and septic shock in the critically ill patients [1]. These infections encompass a range of types, with generalized peritonitis associated with the highest mortality rates [2]. In addition to bacterial pathogens, *Candida* species can also be involved in intra-abdominal infections. Currently, the diagnosis of intra-abdominal candidiasis (IAC) is based on the presence of *Candida* in abdominal samples obtained during surgery [3, 4]. IAC accounts for a substantial proportion of invasive candidiasis in critically ill patients [5], occurring in both hospital- and community-acquired in 13–33% and 7–32% of cases, respectively [6]. *Candida albicans* is the primary causative agent of IAC [7, 8]. The mortality rate associated with IAC ranges from 20 to 50% [2, 9]. However, the reasons behind the high mortality in IAC remain unclear, especially in the absence of candidemia.

While *Candida* is considered a harmless colonizer in the digestive tract, its role as a true pathogen in IAC is still debated [10]. Isolating *Candida* from the peritoneal fluid (PF) without candidemia poses challenges in determining its pathogenicity and its association with mortality [11, 12]. Antifungal treatment studies in IAC have yielded inconsistent results regarding patient survival [13–17]. Some researchers consider the presence of *Candida* as a marker of disease severity [18]. Despite this, there is a lack of studies investigating *Candida* virulence in human PF.

*C. albicans* possesses various virulence mechanisms that enable tissue invasion, evasion of host defenses, and biofilm formation [19, 20]. However, most of these mechanisms have been studied in the context of candidemia, where *Candida* must overcome natural barriers to reach the bloodstream [21]. The course of infection in IAC differs, as the breach in the intestinal wall is caused by anastomosis leakage, necrosis, or perforation. Notably, candidemia is only associated with a minority of IAC cases [7], suggesting alternative triggers for *C. albicans* virulence.

In this study, our hypothesis was that in the context of secondary peritonitis, *C. albicans* develops virulence factors as a means of adapting to the new environmental conditions presented by the peritoneal fluid. In addition, we explored whether this adaptation was influenced by interactions with other microorganisms. Using a step-by-step and multimodal approach, this experimental study aims to evaluate the influence of PF issued from critically ill patients with intra-abdominal infections on *C. albicans* virulence.

## Methods

### Study design and setting

This study was an observational exploratory monocentric study conducted at the Université de Lorraine, in the laboratory “Stress, Immunité et Pathogènes” (SIMPA, UR 7300), and at the University Hospital of Nancy from January 2021 to December 2022. The study was registered with clinicaltrials.gov under the number NCT05264571 (retrospectively registered 22 February 2022). This study was an ancillary study of a non-interventional prospective cohort study (the pBDG2 study, prospective evaluation of 1.3 β-d-glucan in the PF for the diagnosis of intra-abdominal candidiasis in the critically ill patients NCT03997929). The pBDG2 study included critically ill patients with intra-abdominal infections requiring abdominal surgery and allowed the constitution of a biological collection of peritoneal fluid samples.

### Abdominal samples

The samples used in this study consisted of PF obtained from critically ill patients with intra-abdominal infections, and ascitic fluid (AF) obtained from critically ill patients with decompensated cirrhosis of non-infectious origin. AF has the advantage to be the accessible sterile, biological fluid with the closest composition to PF. These samples were obtained after routine analysis, and the remaining samples were selected for use in this study. All selected samples underwent microbiological analysis to detect the presence of bacteria and fungi.

To be eligible for selection, PF samples had to meet the following criteria: absence of *Candida* infection, derived from the initial abdominal surgery (excluding relaparotomy), obtained from the University Hospital of Nancy, and having a minimum volume of 5 mL.

### Strains and media

The SC5314 reference *C. albicans* strain was used for all experiments, as its growth and genome are well-described in the literature [22]. For the evaluation of metabolic production, clinical strains of various bacteria were utilized.

For all experiments (phenotype, molecular, metabolic) three different media were employed: two as controls (Sabouraud and AF) and the PF. Sabouraud medium (SBD) served as a control medium for assessing growth, morphology, and metabolic profile. AF was obtained as a non-infected clinical sample from humans, and its lack of infection was confirmed through microbiological analysis (direct examination, conventional culture) and cytology (neutrophil count < 250/mm^3^). PF, on the other hand, could exhibit different characteristics and might be infected with various bacteria, both positive and negative gram. Since AF and PF were collected consecutively, they were stored at − 20 °C until each experiment period. All analyses were conducted in duplicate or triplicate.

### Phenotypic approach

#### Inoculation of *C. albicans* in the different media

*C. albicans* SC5314 strain was inoculated in triplicate into one mL of the three different types of media (SBD, AF, PF) at an optical density (OD) of 0.3 nm, corresponding to approximately 3.10^6^ colonies of *C. albicans* per millilitre (*C. albicans*/mL) of media. Control wells containing only the media without *C. albicans* were also included on the same 24-well plate to confirm the absence of contamination. All OD measurements were performed at 600 nm with a UV/Visible spectrophotometer P4 from VWR^®^ (Radnor, Pennsylvania, United States).

#### Observation of C*. albicans* in each media

The 24-well plate was inoculated and incubated at 37 °C with shaking at 300 RPM for 24 h. First, the morphology of *C. albicans* was observed under a light microscope at hourly intervals for the initial 8 h, followed by observations at H16 and H24. The analysis of morphology was conducted by ME, in collaboration with the members of the SIMPA laboratory. The results were subsequently validated by MM, an expert in mycology, who was blinded to the sample types. Any discrepancies were resolved through consensus or consultation with a third independent researcher. Second, *C. albicans* growth was assessed by inoculating 10 μL from each well onto SBD dextrose agar, and the colonies were counted after 24 h of incubation at 37 °C. To facilitate counting, 1000-fold dilutions of each inoculated well were prepared beforehand.

#### Composition of ascitic and peritoneal fluid

Each liquid received biochemical measurements (protein, glucose, pH) and cell counts using flow cytometry.

### Molecular approach

*C. albicans* inoculation in the different media followed the same protocol as for phenotypic evaluation, with an overnight culture of 24 h before gene expression analysis.

#### Genes of interest

The primers utilized for reverse transcription and quantitative polymerase chain reaction (RTq-PCR) analysis can be found in supplementary material (Additional file 1: Table S1). The five virulence genes of interest are UME6, ALS3, SFL2, HWP1 and ECE1. Basically, UME6 and SFL2 have a role in *Candida* filamentation. ALS3, HWP1, and ECE1, have a role in adhesion and epithelial cells damage. Please refer to supplementary material for the precise role of each gene.

#### RNA extraction

After the 24-h overnight culture, the samples were centrifuged to retain only the cell pellet. The cell pellet was then washed twice with 10 mL of phosphate-buffered saline (PBS). Subsequently, the PBS was removed, leaving behind only the cell pellet.

RNA extraction was performed using the FASTPREP^®^ lysis technology (MP Biomedicals, Santa Ana, California, United States), following the manufacturer’s program specifically designed for *Candida* cells. To assess the quality and measure the RNA concentration, each sample was analyzed using a Nanodrop 2000 system (Thermo Scientific, Waltham, MA, United States). Total RNA extraction was carried out using the Monarch Genomic RNA Purification Kit (New England Biolabs^®^ Inc., Ipswich, MA, United States) in accordance with the manufacturer’s instructions.

#### Reverse transcription

Reverse transcription was performed using the QuantiTect Reverse Transcription kit from Qiagen^®^ (Germantown, Maryland, United States) following the manufacturer's instructions. The samples were prepared in two steps: genomic DNA removal and reverse transcription. The reverse transcription process was carried out using a thermocycler, following the specified protocol. The steps involved were as follows: annealing at 25 °C for 3 min, reverse transcription at 45 °C for 10 min, and inactivation at 85 °C for 5 min.

#### Amplification by PCR

The qPCR was performed in MicroAmp Optical 96-Well Reaction Plates (Applied Biosystems) using the CFX96 Real-Time PCR System (Bio-Rad, Marnes-la Coquette, France). Please refer to supplementary material **S1** for the details regarding the primers and cycling protocol. Relative transcript levels and the fold change of SFL2, UME6, ALS3, HWP1 and ECE1 were determined following the ΔΔCT method [23]. All analyses were performed in duplicate with negative control samples. The level of expression was compared among the PF against AF level expression, to ensure the comparability in a clinical sample.

### Metabolic approach using the calscreener™ technology

#### The calscreener™ technology

**Fig. 1:**
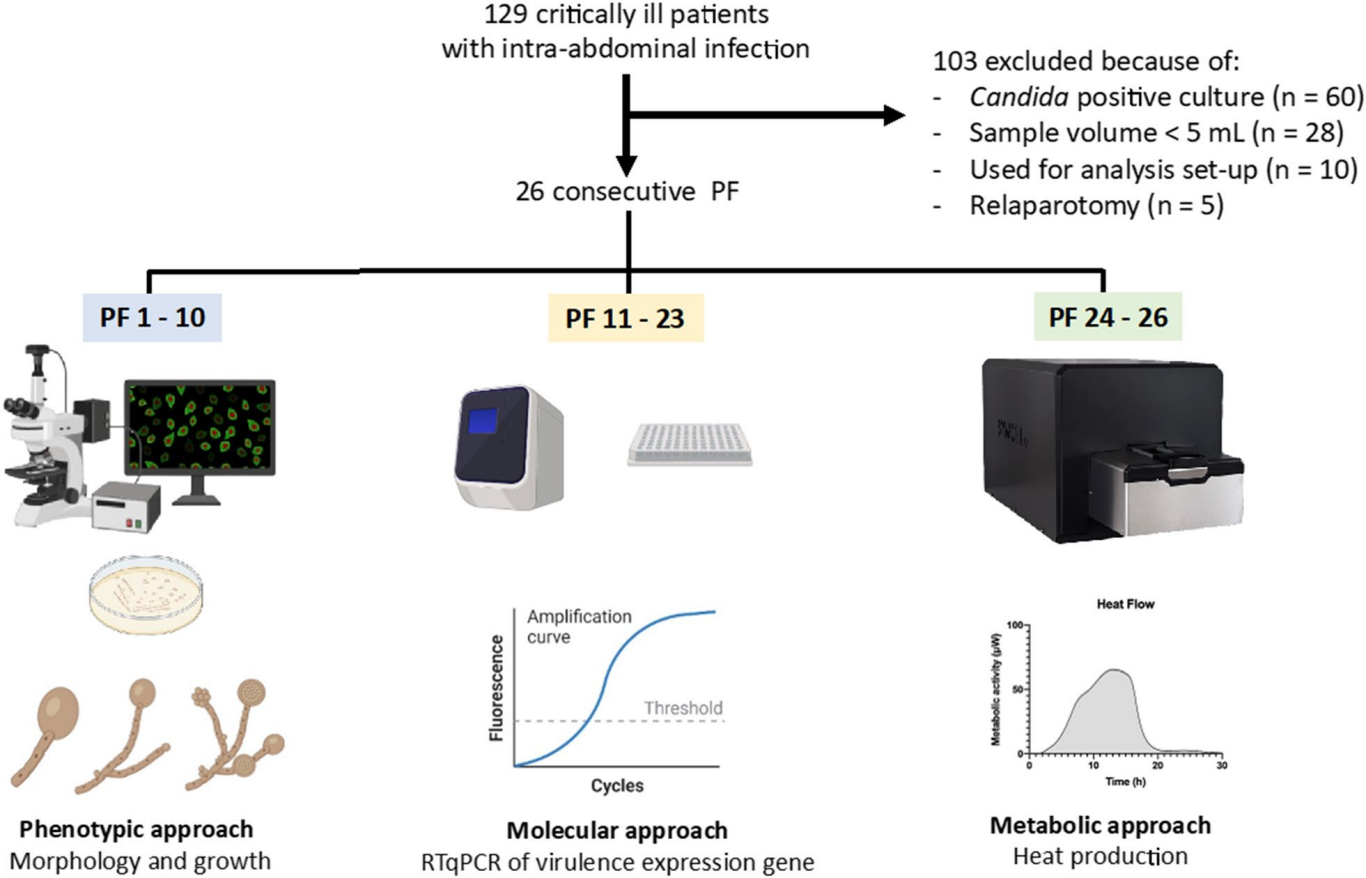
Peritoneal fluid selection and type of analysis. Metabolic approach has been performed with the calscreener™, picture of the equipment was reproduced with permission from Symcel AB (Solna, Sweden). PF: peritoneal fluid; RTqPCR: reverse transcription quantitative polymerase chain reaction

The metabolic evaluation was conducted using the calScreener™ technology (Symcel AB, Solna, Sweden) (Fig. 1) [24]. This technology utilizes isothermal calorimetry to measure the heat flow generated by living cells in real time. It can be applied to various microorganisms and media types. The calScreener™ device provides continuous real-time data over an extended duration. Different parameters can be evaluated directly from the curve, such as time to activity, time to peak or decay time. Analysis of the heat production is carried out using the dedicated software, calView^®^ (Symcel AB, Solna, Sweden). Each specific pathogen generates a distinct growth pattern in the kinetic data, enabling identification of the pathogen type [25]. This technology utilizes 48-well microtiter cell culture plates, accommodating 32 biological samples and 16 thermodynamic internal reference positions.

#### Metabolic analysis

For the inoculation of *C. albicans* in the different media, the same protocol was followed as for the phenotypic and molecular evaluation. The overnight culture of *C. albicans* for 24 h was used before transferring the samples into the calScreener™. It should be noted that all AF and PF samples used were confirmed to be free of pathogens to serve as controls for the added inoculum. Each culture was then appropriately diluted, either 1/100 or 1/1000, in each medium as per the recommendations provided by Symcel. Subsequently, 200 µL of each medium, in duplicate, were transferred to the calScreener™ sample handling system. Real-time measurement of the heat produced from each calWells was carried out at 37 ± 0.001 °C in the calScreener™ up to 48 h. The presence of an active pathogen is associated with a metabolic activity > 5 microW, according to the manufacturer. The remaining samples in the microtitre plate were incubated at 37 °C for 24 h to ensure the presence of expected pathogens.

### Statistical analysis

Due to the exploratory and observational nature of this study, a predetermined calculation of the required sample size was not performed. The sample size was based on the availability of PF samples during the study period. Descriptive statistics were computed using GraphPad Prism version 9 (Boston, Massachusetts, United States). The statistics included counts, means, standard deviation, medians, quartiles, and interquartile ranges (IQR), as deemed appropriate for the analysis.

To compare the heat production between samples during the metabolic evaluation, ordinary one-way ANOVA and Mann–Whitney test were employed. This statistical analysis was used to assess any significant differences in heat production among the samples. A *p* value < 0.05 was considered significant.

## Results

During the designated study period, a total of 129 critically ill patients who underwent intra-abdominal surgery were enrolled as participants. Among these patients, 26 PF samples met the eligibility criteria for inclusion in the study. Details regarding patient characteristics are provided in Table 1. PF characteristics’ (macroscopic aspect and bacterial culture) are provided in Table 2 and Additional file 1: Table S2. Overall, patients with intra-abdominal infections were mostly male [*n* = 19 (73%)] with a median age of 58 years admitted for urgent surgery [*n* = 20 (78%)]. All the PF samples were associated with cases of secondary peritonitis, including 13 post-operative peritonitis (50%). The infected PF samples predominantly exhibited polymicrobial composition. The majority of patients from whom AF samples were collected were post-operative decompensated cirrhosis [*n* = 6 (60%)].

**Table 1:** Characteristics of critically ill patients sampled for peritoneal and ascitic fluids

	Peritoneal fluid	Ascites
Variable	*N* = 26	*N* = 10
*Demographic data*		
Sex (Male)	19 (73)	7 (70)
Age (years)	58 [52–77]	59 [46–66]
Body mass index (kg/m^2^)	26 [23–32]	28 [25–33]
Mac Cabe 1/2/3	10 (38)–14 (54)–2 (8)	1 (10)–7 (70)–2 (20)
Knauss A/B/C	3 (11)–14 (54)–9(35)	0–5 (50)–5 (50)
*Comorbidities*		
Cardio-vascular disease	5 (19)	1 (10)
Chronic obstructive pulmonary disease	3 (11)	1 (10)
Diabetes	9 (35)	5 (50)
Chronic kidney disease	5 (19)	3 (30)
Immunosuppression condition Organ or bone-marrow transplantation Solid tumour (active) Haematological malignancy Systemic inflammatory disease	12 (46) 2 (16) 7 (58) 1 (8) 2 (16)	4 (40) 4 (100)
Cirrhosis A/B/C	0	3 (30)–3 (30)–4 (40)
*Intra-abdominal infection*		
Secondary peritonitis	26 (100)	NA
Hospital-acquired Post-operative peritonitis Community-acquired	21 (81) 13 (50) 5 (19)	NA
Anatomical source		NA
Stomach Biliary tract Pancreas Small bowel Colon	4 (15) 2 (8) 2 (8) 6 (23) 12 (46)	NA
*ICU data*		
Type of ICU admission		
Medical Scheduled surgery Urgent surgery	3 (11) 3 (11) 20 (78)	4 (40) 6 (60) 0
Admission SAPS II score	46 [31–57]	49 [47–56]
IAI SOFA score	6 [4–]	NA
Septic shock (Sepsis-3 definition)	14 (54)	2 (20)
Norepinephrine infusion Norepinephrine duration (days)	25 (96) 2 [1–3]	5 (50) 4 [4–9]
Invasive mechanical ventilation (> 48 h) Duration of invasive mechanical ventilation (days)	8 (31) 3.4 ± 6.3	4 (40) 9.8 ± 5.6
Renal failure (according to KDIGO—AKI definition) Renal replacement therapy	17 (65) 4 (15)	7 (70) 2 (20)
ICU length of stay (days)	7 [5–12]	10 [5–19]
ICU mortality	5 (19)	3 (30)

Data are presented as Median [interquartile range] or number (percentage) or mean ± SD

NA: not applicable. AKI: Acute Kidney Injury; IAI: Intra-abdominal infection; ICU: Intensive Care Unit; SAPS: Simplified Acute Physiology Score; SOFA: Sequential organ failure assessment

**Table 2:** Bacterial composition, macroscopic examination of the peritoneal fluid, and observed morphology and growth of *C. albicans* in peritoneal fluids 1 to 10

Characteristics	PF-1	PF-2	PF-3	PF-4	PF-5	PF-6	PF-7	PF-8	PF-9	PF-10
Germs identified in routine microbiology	*S. constellatus* *E. coli* *B. fragilis*	*Poly-microbial flora*	*S. anginosus*	*E. coli*	*P. aeruginosa* *E. faecalis*	*E. coli* *P. vulgaris*	*E. coli* *K. pneumoniae*	*M. morganii* *E. coli* *E. faecalis*	*Poly-microbial flora*	*E. faecalis*
Macroscopic examination of PFF	Fecal	Fecal	Yellow	Bloody	Yellow	Yellow	Yellow	Yellow	Bloody	Yellow
*C. albicans*/ml at H0	3 030 000	1 570 000	3 125 000	2 650 000	3 040 000	3 115 000	3 265 000	3 000 000	3 100 000	3 955 000
*C. albicans*/ml at H24	181 800	32 060 000	14 875 000	9 081 000	4 986 000	3 177 000	816 000	28 000 000	15 400 000	3 283 000
Ratio of *C. albicans*/ml at H24 and H0	0.06	20.42	4.76	3.43	1.64	1.02	0.25	9.33	4.97	0.83
Microscopic examination of *C. albicans* at H24	Yeast	Pseudohyphae		Hyphae without conglomerates				Hyphae with conglomerates		

Different PF samples were utilized for the three distinct approaches due to the step-by-step nature of the study, limited volume of collected samples per patient, and availability of the calScreener™.

Figure 1 provides a visual representation of the sample selection and allocation process for the three different analyses conducted in the study.

### Phenotypic approach

**Fig. 2:**
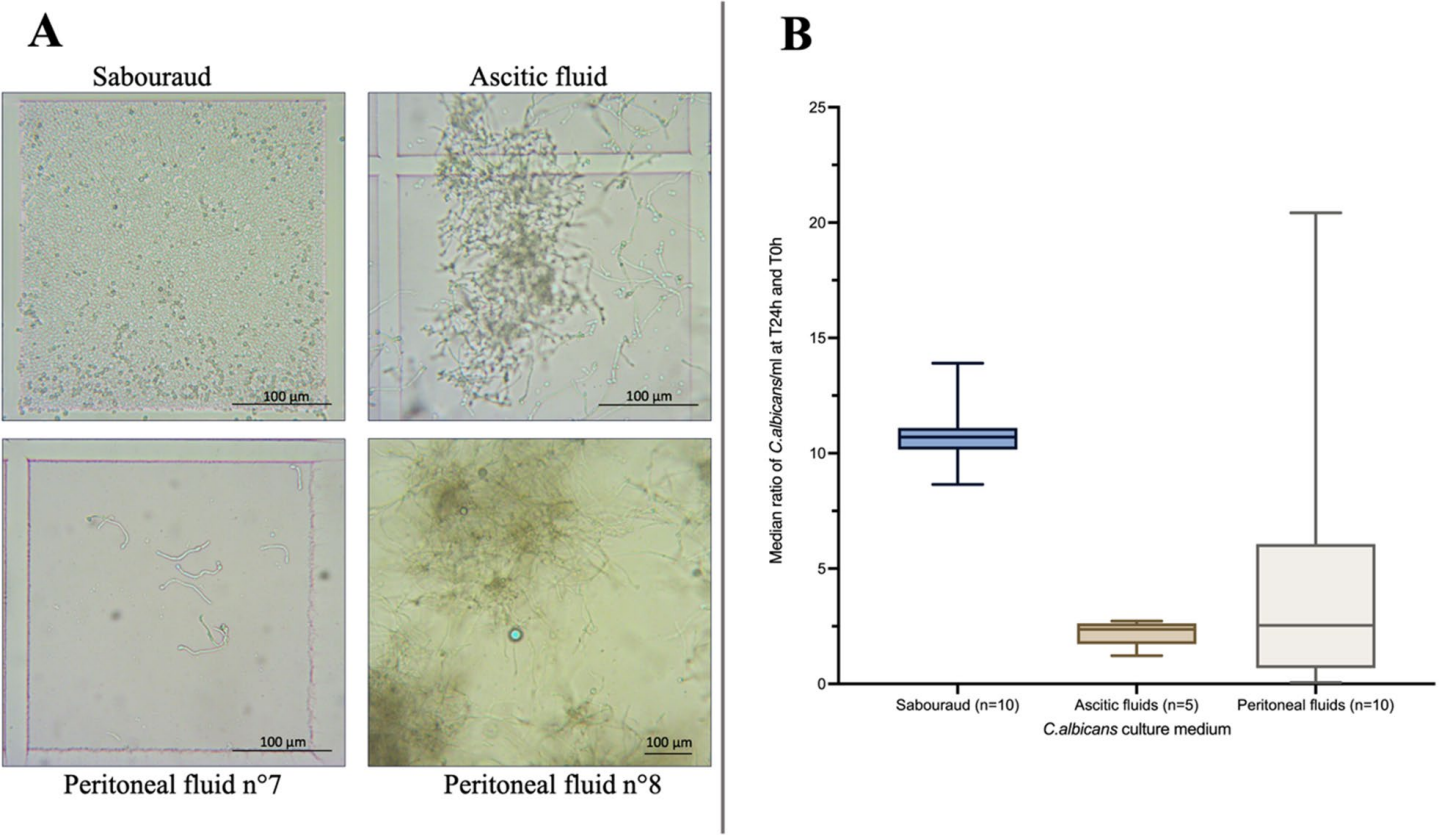
Phenotypic approach: morphology and growth of *C. albicans* according to the media. **A** morphology of *C. albicans* depending on the media, after 24 h. **B** Growth of *C. albicans* depending on the media, after 24 h (Box plot showing median, interquartile range and min–max value). More images are available in the supplementary materials (Additional file 1: Figure S1)

Figure 2A illustrates the morphology of *C. albicans* in the different media used in the study. The SBD medium served as the control for each plate layout. No contamination was observed. The microscopic examination of *C. albicans* after growth in SBD (*n* = 10) revealed a consistent presence of unicellular ovoid yeast forms without any hyphae. This morphology remained unchanged throughout the various manipulations.

After inoculating *C. albicans* from the SBD wells onto SBD dextrose agar, the colony count results demonstrated a consistent increase in the number of *C. albicans* colonies from H0 to H24, as shown in Fig. 2B. At H0, the median number of colonies of *C. albicans* per mL at H0 was 3,010,000 (*n* = 10, IQR = 2.5), which then increased to 32,150,000 (*n* = 10, IQR = 173.2) at H24. This indicates a 10.7-fold median increase in the number of *C. albicans* colonies from H0 to H24 (IQR = 0.44).

In AF, no contamination was observed. The microscopic examination of *C. albicans* after growth in AF revealed a transition from oval yeast forms to filamentous hyphae forms. The hyphae tended to form conglomerates. After inoculating *C. albicans* from AF wells onto SBD dextrose agar, the colony count results showed a consistent increase in the number of *C. albicans* colonies from H0 to H24, as depicted in Fig. 2B. The median number of *C. albicans* colonies per mL at H0 was 2,770,000 (*n* = 5, IQR = 25.0), which then increased to 7,500,000 (*n* = 10, IQR = 94.0) at H24. This represents a 2.37-fold median increase in the number of *C. albicans* colonies from H0 to H24 (IQR = 0.28).

In PF, no contamination was observed. Table 2 provides an overview of the phenotypes of *C. albicans* observed in PF samples 1 to 10. The microscopic examination of *C. albicans* after growth in PF exhibited variability, with observations of yeasts alone, hyphae, and both with and without conglomerates (Fig. 2A). After inoculating *C. albicans* from PF wells onto SBD dextrose agar, the colony count results demonstrated variable changes in the number of *C. albicans* colonies from H0 to H24 (Fig. 2B). For instance, the number of *C. albicans* colonies per mL could be up to 21 times higher at H24 compared to H0 in PF2, while in PF7, the number decreased between H0 and H24. The median number of *C. albicans* colonies per mL at H0 was 2,557,000 (*n* = 10, IQR = 155), and at H24, it was 2,325,000 (*n* = 10, IQR = 309.87). In summary, there was a 1.48-fold median increase (*n* = 10, IQR = 3.11) in the number of *C. albicans* colonies per mL from H0.

#### Composition of fluid used for the phenotypic approach

In case of PF (*n* = 10/10), we noted a median/IQR count of leucocytes of 42.9 [13.2–72.8] G/L mostly consisting of neutrophils (median 39.0 [13.0–72.9] G/L), a median protein of 38.0 [29.7–44.7] g/l, a median glucose of 0.30 [0.22–0.47] mmol/L, and a median pH of 8.50 [8.25–8.67]. As for AF (*n* = 5/10), the median count of leucocytes was 0.12 [0.08–0.16] G/L, the median protein was 12.0 [12.0–17.0] g/l, the median glucose was 6.50 [5.9–7.9] mmol/L, and the median pH was 7.50 [7.50–7.70]. For a comprehensive breakdown of these measurements, refer to the Additional file 1: Table S3.

### Molecular approach

**Fig. 3:**
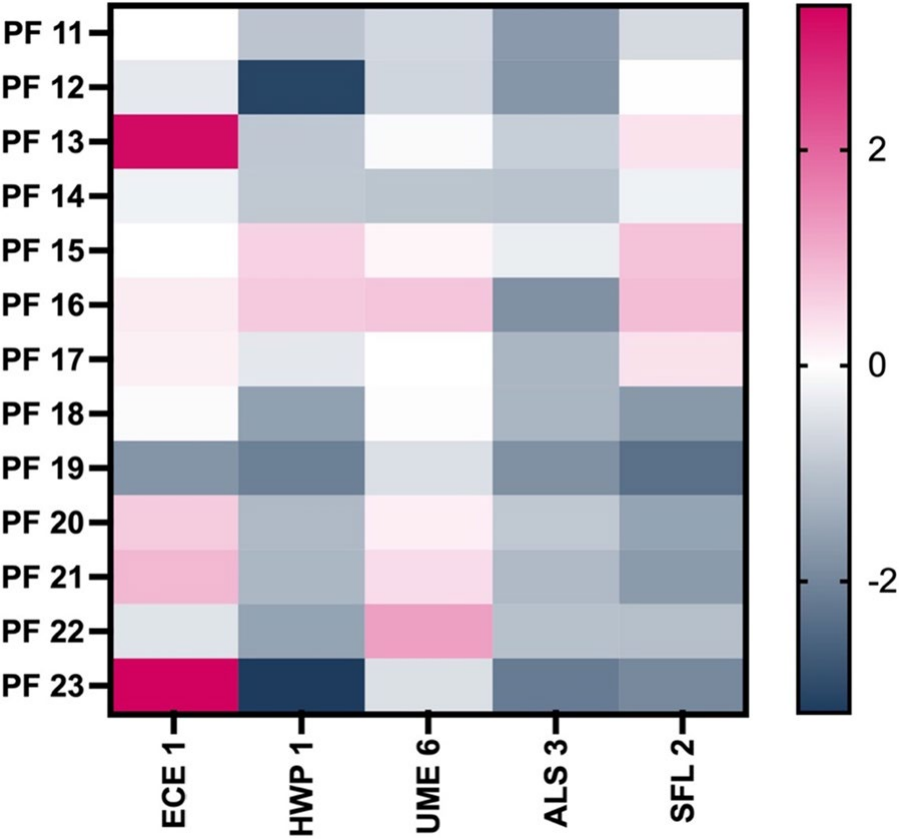
Heat map of virulence expression gene in PF 11 to 23. Legend: heat map showing log-transformed quantitative genes expression of *C. albicans* grown in different peritoneal fluids. Gene expression of *C. albicans* in peritoneal fluids is compared with ascitic fluid. Relative gene expression was measured by RTqPCR using the ΔΔCT method. PF: peritoneal fluid

The expression of *C. albicans* genes was analysed and log-transformed in 13 different PFs. Figure 3 depicts the heatmap of ECE1 HWP1, UME6, ALS3, and SFL2 expression in the different PFs compared to those in AF. The heat map showed the highly variable expression of these five genes, reflecting the different impacts of each PF on *C. albicans* gene expression.

### Metabolic approach

**Fig. 4:**
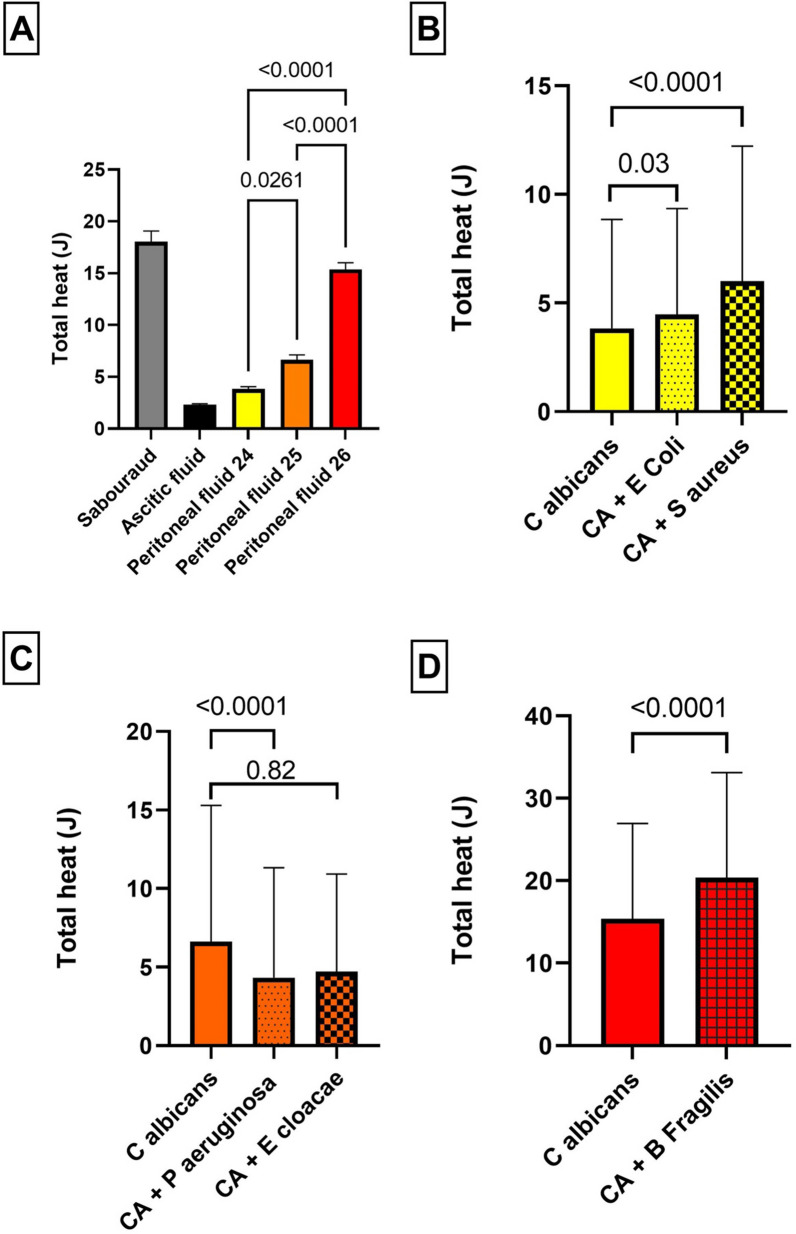
Heat production of *C. albicans* alone and combined with different bacteria, according to the media. Heat production is expressed as mean and standard deviation. **A** Total heat production after 48 h of *C. albicans* depending on the type of media (Sabouraud, ascitic fluid, peritoneal fluid number 24–26). **B–D** Total heat production after 48 h of *C. albicans* with different bacteria (**B** *Escherichia coli*, *Staphylococcus aureus* in PF24; **C** *Pseudomonas aeruginosa*, *Enterobacter cloacae* in PF25; **D** *Bacteroides fragilis,* in PF26). PF: peritoneal fluid

In Fig. 4A, the total heat production was compared between different media when the same amount of *C. albicans* was inoculated alone. It was observed that SBD medium produced the highest amount of heat, with a mean of 18.03 J, which was statistically significant compared to other media (*p* < 0.0001). The heat production in AF was lower than in PF, and there was a statistically significant difference between AF and PF25 as well as PF26 (*p* < 0.001). Among the three different PFs evaluated, there was also a statistically significant difference in heat production: PF24 had a mean of 3.83 J, PF25 had a mean of 6.62 J, and PF26 had a mean of 15.37 J (*p* = 0.0261, *p* < 0.0001, *p* < 0.0001).

In Fig. 4B–D, the heat production was analysed for samples combining *C. albicans* and bacteria. Apart from one combination (PF25, *C. albicans* with *E. cloacae*), the differences in metabolic production between *C. albicans* alone and in conjunction with bacteria were statistically significant across all other combinations. These associations yielded both increased metabolic rates (notably with *E. coli*, *S. aureus*, and *B. fragilis*) and decreased metabolic rates (particularly with *P. aeruginosa* and *E. cloacae*).

In terms of comparing pathogen associations within the same PF (Additional file 1: Figure S2), the combination of *C. albicans* with *S. aureus* generated a higher heat production than the combination of *C. albicans* with *E. coli* (*p* < 0.01) in PF24. Similarly, in PF25, the combination of *C. albicans* with *E. cloacae* resulted in higher heat production than the combination of *C. albicans* with *P. aeruginosa* (*p* < 0.05).

The difference between samples heat production, time to activity, time to peak, time to decay, metabolic activity between samples and pathogen combination are described in supplementary materials (Additional file 1: Table S4, Figure S3). No contamination and survival of all involved yeast and bacterial strains were confirmed after inoculation of the remaining sample on culture plates.

## Discussion

This study represents the first reported investigation into the expression of *C. albicans* virulence in human peritoneal fluid using three distinct approaches that consistently yielded similar results. The findings of this study could challenge the notion that *C. albicans* should be solely regarded as a harmless colonizer in PF and highlight its potential pathogenic role. Moreover, the study demonstrates that the expression of *C. albicans* virulence factors in PF seems to be highly variable and influenced by the specific characteristics of the fluid as well as the presence of bacteria.

The phenotype approach confirmed the presence of hyphae forms of *C. albicans* in the PF. The ability of *C. albicans* to transition from yeast to hyphal forms is considered a crucial virulence attribute [20]. This transition confers *C. albicans* with the potential to penetrate epithelial and intestinal barriers and evade the immune system [19] which are well-established mechanisms in candidemia [26]. However, in the present study, the presence of hyphal forms was observed in the PF without the requirement of barrier crossing. The switch to hyphal forms is known to be triggered by various factors, including interactions with bacteria, immune cells, and environmental conditions, such as temperature and pH [27–29]. The variability in the composition of PF may explain the observed variation in *C. albicans* forms.

In this context, it remains challenging to pinpoint specific parameters within the PF that are accountable for the observed variability. The composition of the PF in secondary peritonitis predominantly featured a significant presence of immune cells, primarily neutrophils, alongside relatively elevated protein levels and diminished glucose concentrations. Despite our efforts, we were unable to detect a discernible pattern in the PF composition that correlated with a higher occurrence of virulence expression. This could be attributed to the limitations imposed by the sample size. Consequently, the need for further investigations becomes apparent, necessitating a larger sample pool and a more comprehensive analysis that encompasses factors, such as the specific types of proteins (such as immunoglobulins), sources of carbon (for instance, lactate), and the immune components (comprising cell types and cytokines) at play. In addition, some PF samples exhibited a mixture of hyphal and yeast forms, a phenomenon previously reported. The yeast form is believed to facilitate dissemination, while the hyphal form is associated with tissue damage [30].

The evaluation of different genes involved in the mechanisms of virulence in *C. albicans* revealed that the hyphal switch is not the sole mechanism contributing to its virulence [31]. For instance, HWP1, an adhesin gene, is essential for mucosal epithelial cell adhesion and biofilm formation [32]. ECE1 is essential for the synthesis of candidalysin, a cytolytic peptide toxin that directly damages host epithelial membranes [33]. Molecular analyses confirmed the observations made during the phenotypic experiments, indicating that the expression of these five *C. albicans* virulence genes was highly variable depending on the composition of the PF. The PF components appear to influence both the phenotype of *C. albicans* and the expression of its virulence genes.

The metabolic analyses conducted in the study revealed significant variability in heat production depending on the composition of the PF and the presence of bacteria. Heat production is known to be associated with microbial growth and activities, and it is considered to be more accurate and sensitive compared to standard culture methods [34, 35]. Interactions between *Candida* and bacteria have been previously demonstrated [36]. In animal model, the intra-peritoneal co-injection of *C. albicans* and *Staphylococcus aureus* led to higher mortality in mice compared to single infections [37]. Co-infection with *E. coli* and *C. albicans* resulted in higher mortality in mice compared to mono-infection [38]. Regarding heat production, the presence of bacteria yielded diverse outcomes in terms of metabolic rates. Combinations with *E. coli*, *S. aureus*, and *B. fragilis* led to heightened metabolic rates, while pairings with *P. aeruginosa* and *E. cloacae* resulted in reduced metabolic rates. Analysing the area under the curve of metabolic production (refer to Additional file 1: Figure S3), only the association with *B. fragilis* demonstrated a sustained signal at 24 h, indicating a potential synergistic activity that might be attributed to biofilm formation. Previous experimental studies have demonstrated that *C. albicans* contributes to the survival of *B. fragilis* through biofilm formation, creating an anaerobic microenvironment that promotes the growth and persistence of *B. fragilis* [39].

The findings of this study are consistent with two recent animal studies conducted in mice, which also explored the influence of PF on the virulence of *C. albicans* [40, 41]. Lima et al*.* reported that the presence of fecal material in the peritoneal cavity reduced the invasiveness of *C. albicans* [40]. Cheng et al. demonstrated that the PF composition, including local pH and molecular signatures, influenced the expression of virulence genes, indicating a role in modulating *C. albicans* virulence during abscess formation [41].

Major strength of this study is its unique approach in assessing the pathogenicity of *C. albicans* using PF obtained from human intra-abdominal infections, which provides more relevant insights compared to animal models, which have certain limitations. For instance, these models often involve immunosuppressed mice, which may not fully represent the immune status of human patients with intra-abdominal infections. In addition, the administration of *C. albicans* through intra-peritoneal injection in animal models does not replicate the complete spectrum of pathogenesis observed in human cases, including the interactions between the gut microbiota and the host before the breach of the intestinal barrier. Finally, the immune, inflammatory, and metabolic responses to infection in mice differ significantly from the human phenotype of sepsis [42].

### Limitations

We acknowledge limitations in our study. First, the sample size was relatively small, and we were unable to perform combined analyses using the triple approach on the same sample. This limitation arose due to the stepwise approach of the study, limited sample volume, and restricted access to the calScreener™ equipment. Second, the extraction of RNA from human PF was particularly challenging and had not been previously attempted. Overcoming these technical difficulties was a notable achievement of our study. Finally, the use of the calScreener™ for PF and *Candida* species was a novel application. Indeed, previous studies have been conducted using the calScreener™ equipment with bacteria [25], but its application to PF or *Candida* species has not been explored before. We had to dedicate some PF samples to test its feasibility and reliability, as well as to configure the inoculum accurately for ensuring comparability between samples.

This study was observational, and it revealed a high variability in the expression of *C. albicans* virulence and metabolic profiles. Additional investigations are necessary to uncover the precise factors contributing to the variation in *C. albicans* virulence expression. While bacteria were introduced as a modifiable element, it remains challenging to pinpoint the exact constituents of the peritoneal fluid responsible for driving this virulence expression.

It is important to note that this study focused specifically on *C. albicans*, and the results may not be applicable to other *Candida* species with different virulence mechanisms, such as *C. glabrata*. In addition, the study only examined samples from secondary peritonitis, limiting conclusions regarding primary and tertiary peritonitis. Spontaneous fungal peritonitis is a primary peritonitis which shares commonalities with IAC. Of note, the treatment necessity, especially in cirrhotic patients with acute-on-chronic liver failure is debated [43, 44]. Spontaneous fungal peritonitis necessitates a translocation of *Candida* across the gastrointestinal tract mucosa into the peritoneal cavity, a process exacerbated by factors, such as immunosuppression or malnutrition. The evaluation of virulence expression of *Candida* during spontaneous fungal peritonitis may help to address the question regarding treatment.

Certainly, the next step would involve assessing the virulence of clinical strains of *Candida* obtained from critically ill patients with IAC and correlating the expression of virulence factors with clinical outcomes. However, before conducting these analyses, further optimization is necessary for the molecular and metabolic evaluations. RNA extraction from PF proved to be challenging, as did ensuring an adequate quantity of inoculum for metabolic assessment. One advantage of this experimental study was the ability to control both the inoculum and the experimental conditions.

## Conclusion

This study provides the first evidence of *C. albicans* virulence in human peritoneal fluid. The expression of *C. albicans* virulence was found to be highly variable in PF samples from critically ill patients with intra-abdominal infection. These findings could challenge the current understanding of IAC, which primarily relies on clinical signs of infection and positive *Candida* cultures. Further research is needed to identify the specific components of PF that promote *C. albicans* virulence. The next important step is to determine the factors that trigger the transition of *Candida* from a harmless colonizer to a life-threatening pathogen, allowing for targeted treatment in critically ill patients with invasive candidiasis. In addition, there is a need for studies investigating the immune response in the context of IAC involving the human population. Indeed, the ability of the immune system to clear the peritoneum from pathogen including *Candida* is of high interest to understand clinical outcomes, such as mortality. Besides, *Candida* can escape the immune system among other with hyphae transformation and biofilm formation. Thus, to better understand changes in *Candida* virulence, all components (media, bacteria, immunity) must be evaluated. These studies will contribute to the development of personalized medicine approaches for managing invasive candidiasis in critically ill patients.

### Supplementary Information

**Additional file 1.** Provides the qPCR protocol for the expression of *C. albicans* virulence gene with details regarding each genes (TableS1), the bacterial composition and macroscopic examination of the peritoneal fluids 11 to 26 (Table S2), the cytology, protein and glucose concentrations of included PF 1 to 10 and AF 1 to 5 (Table S3), additional metabolic parameters depending on the peritoneal fluid (24 to 26) and the presence of bacteria (Table S4), enlarged photo from the phenotypic approach (Figure S1), and the heat production profile of *C. albicans* combined with different bacteria in different peritoneal fluids (Figure S2).

## Data Availability

The data that support the findings of this study are available from the corresponding author, but some restrictions apply to the availability of these data, which were used under license for the current study (calScreener™), and so are not publicly available. Data are, however, available from the authors upon reasonable request and with permission of all authors.

## Abbreviations

ACT: Actin
ALS: Agglutinin-like sequence
AF: Ascitic fluid
ECE: Extent of cell elongation
HWP: Hyphal wall protein
IAC: Intra-abdominal candidiasis
ICU: Intensive care unit
IQR: Interval quartile range
OD: Optical density
PBS: Phosphate-buffered saline
PF: Peritoneal fluid
RTqPCR: Reverse transcriptase quantitative polymerase chain reaction
SBD: Sabouraud medium
SIMPA: Stress, Immunité, Pathogènes
TEF: Translation elongation factor
