# Supplementary material for: Reappraisal of intra-abdominal candidiasis: insights from peritoneal fluid analysis
Source: Intensive Care Med Exp. 2023 Sep 30;11:67. doi: 10.1186/s40635-023-00552-0 (PMC10542081; doi:10.1186/s40635-023-00552-0)
Supplement: Supplementary file 1 — Additional file 1. Provides the qPCR protocol for the expression of C. albicans virulence gene with details regarding each genes (TableS1), the bacterial composition and macroscopic examination of the peritoneal fluids 11 to 26 (Table S2), the cytology, protein and glucose concentrations of included PF 1 to 10 and AF 1 to 5 (Table S3), additional metabolic parameters depending on the peritoneal fluid (24 to 26) and the presence of bacteria (Table S4), enlarged photo from the phenotypic approach (Figure S1), and the heat production profile of C. albicans combined with different bacteria in different peritoneal fluids (Figure S2). [file 40635_2023_552_MOESM1_ESM.docx]

**Supplementary material**

**Table S1**. Forward and reverse primers used for qPCR, according to the gene.

| Target gene | Sequence 5’ -> 3’ |
| --- | --- |
| UME6 | FW – TGGTAATGGCACTAACACCAA  RV – CCAAATTTAGCACAACCTCCA |
| ALS3 | FW – ATTCTGAATTTACTACTTCCACAGC  RV – ATGATAGGCGATGAAGCTTC |
| SFL2 | FW – CAGCATCAGCTTTATCTTCC  RV – ACGATAGTTGGTTGAATTCA |
| HWP1 | FW – CTCCAAAATCATCAGCTC  RV – CACTAGCCAAAACAGAAG |
| ECE1 | FW – CACTGGTGTTCAACAATCCAT  RV – AGCATTTTCAATACCGACAG |
| ACT1 | FW – TATGAAAGTTAAGATTATTGCTCCACCAGAAA  RV – GGAAAGTAGACAATGAAGCCAAGATAGAAC |
| TEF 3 | FW – GATCACAATTGGGTCCAAGG  RV – AGCAGCGGCAATCTTGTTAC |

Legend: ACT1: Actin 1; ALS3: agglutinin-like protein 3; ECE1: extent of cell elongation protein 1; FW: Forward primer; HWP1: Hyphal wall protein 1; RV: Reverse primer; TEF3: translation elongation factor 3.

**qPCR protocol:**

The reaction mixtures contained 2 µL of primers (forward and reverse at the concentration of 400 nM of each primer), 7 µL of water, 10 µL of SYBR and 1 µL of cDNA in a final volume of 20 µL.

qPCR was performed in MicroAmp Optical 96-Well Reaction Plates (Applied Biosystems) using the CFX96 Real-Time PCR System (Bio-Rad, Marnes-la Coquette, France) and used the following cycling protocol: polymerase activation during one cycle of 3 min at 98 °C, followed by a denaturation of 15 sec at 95 °C, then by 40 cycles of extension of one minute at 65 to 95 °C (0,5 °C increments at two to five seconds by step).

**Detailed of evaluated gene:**

The UME6 gene is a master regulator of filamentation and is specifically important for hyphal extension [1].

ALS3 is a cell wall adhesin protein that allows *C. albicans* to adhere, invade, and damage epithelial cell [1].

SFL2 is a transcription factor that plays a role as an activator of filamentous growth and is involved in tissue invasion [2].

HWP1 is an important adhesin for developmental regulation and an essential gene for mucosal epithelial cell adhesion and biofilm formation [3].

ECE1 is a gene encoding a membrane protein that is closely related to hyphal extension [3], and is essential for the synthesis of candidalysin, a cytolytic peptide toxin that directly damages host epithelial membranes [4].

ACT1 and TEF3 are the control and calibrator genes, respectively [5].

1. Chen H, Zhou X, Ren B, Cheng L (2020) The regulation of hyphae growth in Candida albicans. Virulence 11:337–348. https://doi.org/10.1080/21505594.2020.1748930

2. Znaidi S, Nesseir A, Chauvel M, et al (2013) A comprehensive functional portrait of two heat shock factor-type transcriptional regulators involved in Candida albicans morphogenesis and virulence. PLoS Pathog 9:e1003519. https://doi.org/10.1371/journal.ppat.1003519

3. Fan Y, He H, Dong Y, Pan H (2013) Hyphae-specific genes HGC1, ALS3, HWP1, and ECE1 and relevant signaling pathways in Candida albicans. Mycopathologia 176:329–335. https://doi.org/10.1007/s11046-013-9684-6

4. Allert S, Förster TM, Svensson C-M, et al (2018) Candida albicans-Induced Epithelial Damage Mediates Translocation through Intestinal Barriers. mBio 9:e00915-18. https://doi.org/10.1128/mBio.00915-18

5. Alonso GC, Pavarina AC, Sousa TV, Klein MI (2018) A quest to find good primers for gene expression analysis of Candida albicans from clinical samples. J Microbiol Methods 147:1–13. https://doi.org/10.1016/j.mimet.2018.02.010

**Table S2:** Bacterial composition and macroscopic examination of the peritoneal fluids 11 to 26.

| Peritoneal fluid | Macroscopic examination | Bacterial culture |
| --- | --- | --- |
| 11 | Yellow | Polymicrobial flora |
| 12 | Bloody | Polymicrobial flora |
| 13 | Yellow | *Serratia Marcescens* |
| 14 | Yellow | *Escherichia Coli* |
| 15 | Yellow | Polymicrobial flora |
| 16 | Fecal | *Klebsiella Pneumoniae*  *Bacteroides Thetaiotamicron* |
| 17 | Fecal | Polymicrobial flora |
| 18 | Fecal | *Escherichia Coli*  *Bacteroides Fragilis* |
| 19 | Yellow | *Escherichia Coli*  *Bacteroides Fragilis* |
| 20 | Yellow | *Enterococcus Faecalis* |
| 21 | Yellow | *Morganella Morganii*  *Escherichia Coli*  *Enterococcus Faecalis* |
| 22 | Bloody | Polymicrobial flora |
| 23 | Yellow | *Pseudomonas Aeruginosa*  *Enterococcus Faecalis* |
| 24 | Yellow | Negative |
| 25 | Bloody | Negative |
| 26 | Yellow | Negative |

**Table S3:** Cytology, protein and glucose concentrations of included PF 1 to 10 and AF 1 to 5.

| **Characteristics** | **PF-1** | **PF-2** | | **PF-3** | **PF-4** | **PF-5** | **PF-6** | **PF-7** | **PF-8** | **PF-9** | | **PF-10** |
| --- | --- | --- | --- | --- | --- | --- | --- | --- | --- | --- | --- | --- |
| Leucocytes (G/L)  PNN  Lymphocytes  Monocytes/macrophages | 3.57  3.32  0.04  0.21 | 87.41  87.41  0  0 | | 57.40  52.02  0.68  4.70 | 28.49  26.40  1.90  0.19 | 10.01  0.89  0.11  0.11 | 22.78  0  0  0 | 74.01  74.01  0  0 | 69.41  69.41  0  0 | 86.81  86.81  0  0 | | 1.16  0.78  0.03  0.25 |
| Protein (g/L) | 46 | 38 | | 38 | 32 | 47 | 20 | 41 | 29 | 48 | | 12 |
| Glucose (mmol/L) | 0.3 | 0.5 | | 0.2 | 0.1 | 0.5 | 0.4 | 0.3 | 0.2 | 0.5 | | 0.3 |
| pH | 8.2 | 8 | 8.7 | | 8.9 | 8.5 | 9 | 0.5 | 8.1 | 8.4 | 8.6 | |
|  |  |  | |  |  |  |  |  |  |  | |  |
| **Characteristics** | **AF-1** | **AF-2** | | **AF-3** | **AF-4** | **AF-5** |  | | | | | |
| Leucocytes (G/L)  PNN  Lymphocytes  Monocytes/macrophages | 0.17  0.09  0.03  0.05 | 0.08  0.02  0.01  0.05 | | 0.08  0.03  0.02  0.03 | 0.12  0.03  0.04  0.05 | 0.17  0  0.06  0.11 |  |  |  |  |  |  |
| Protein (g/L) | 12 | 20 | | 10 | 12 | 17 |  |  |  |  |  |  |
| Glucose (mmol/L) | 5.6 | 7.9 | | 8.2 | 6.5 | 5.9 |  |  |  |  |  |  |
| pH | 7.7 | 7 | 8 | | 7.5 | 7.7 |  |  |  |  |  |  |

Abbreviations: PF: peritoneal fluid; AF: ascitic fluid; PNN: polynuclear neutrophils.

**Table S4**. Metabolic parameters depending on the peritoneal fluid and the presence of bacteria.

**Peritoneal fluid 24**

|  | Time to activity (h) | Time to peak (h) | AUC before peak (J) | Decay time (h) |
| --- | --- | --- | --- | --- |
| *C albicans* alone | 2.05 | 5.51 | 0.12 | 7.75 |
| *C albicans* + *S aureus* | 2.96 | 6.63 | 0.15 | 6.83 |
| *C albicans* + *E Coli* | 2.92 | 6.80 | 0.17 | 4.79 |

**Peritoneal fluid 25**

|  | Time to activity (h) | Time to peak (h) | AUC before peak (J) | Decay time (h) |
| --- | --- | --- | --- | --- |
| *C albicans* alone | 1.16 | 2.2 | 0.09 | 19.73 |
| *C albicans* + *P aeruginosa* | 1.24 | 2.32 | 0.11 | 5.31 |
| *C albicans* + *E cloacae* | 1.08 | 1.78 | 0.10 | 6.55 |

**Peritoneal fluid 26**

|  | Time to activity (h) | Time to peak (h) | AUC before peak (J) | Decay time (h) |
| --- | --- | --- | --- | --- |
| *C albicans* alone | 3.21 | 23.26 | 0.81 | 18.34 |
| *C albicans* + *B fragilis* | 3.27 | 21.20 | 0.93 | 21.91 |

Abbreviations: AUC: area under the curve; h: hours; J: joules

**Legend:**

These parameters give a better overview of the metabolic activity changes depending on the peritoneal fluid and presence of bacteria.

Time to activity corresponds to the time before the first detection of metabolic activity (> 0.005 Joule).

Time to peak (hours) corresponds to the time to obtain the peak of metabolic activity.

Decay time (hours) corresponds to the time to reach no activity after the peak of metabolic activity.

**Figure S1:** Phenotypic approach: morphology and growth of *C. albicans* according to the media.


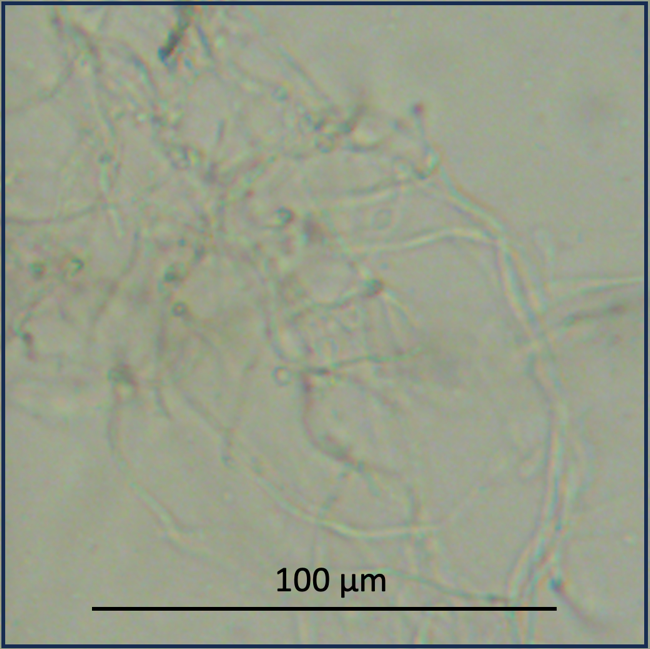

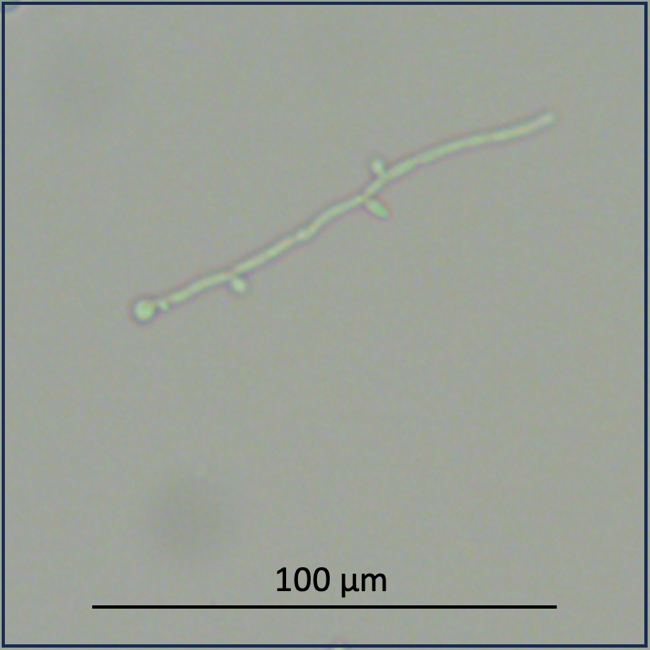

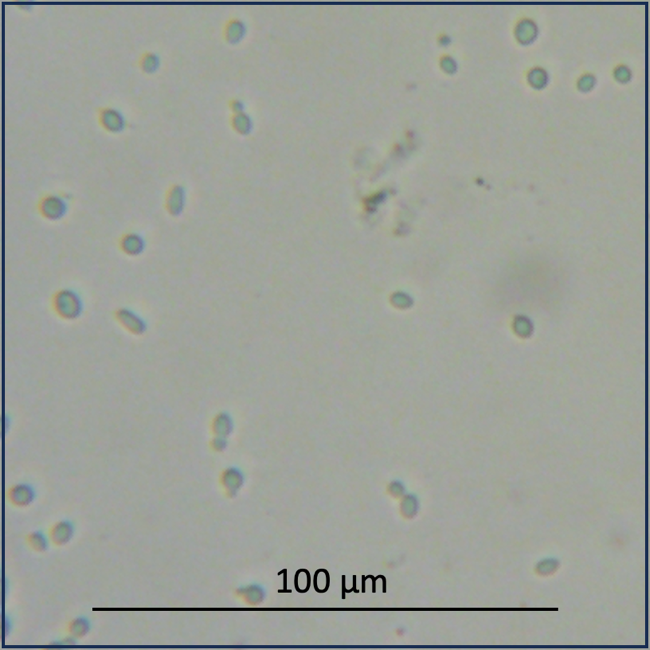

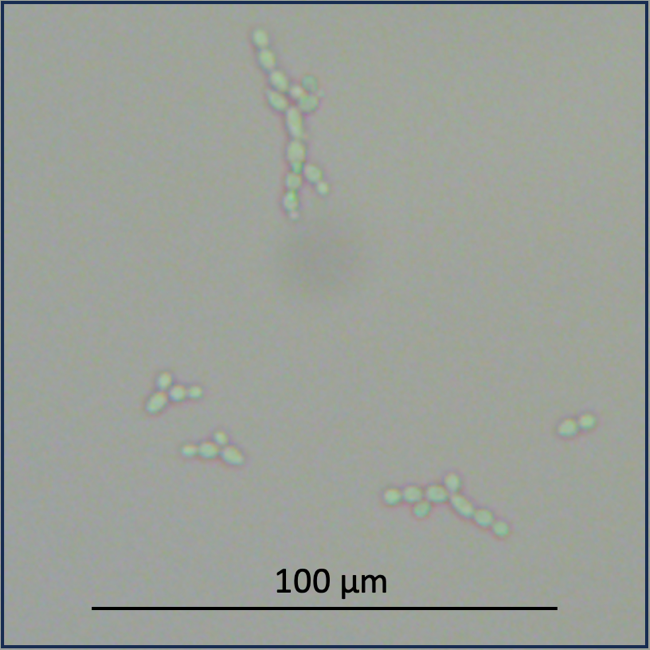


Hyphae with conglomerates, peritoneal fluid n°9

Hyphae without conglomerate, peritoneal fluid n°4

Yeast, peritoneal fluid n°1

Pseudohyphae, peritoneal fluid n°2


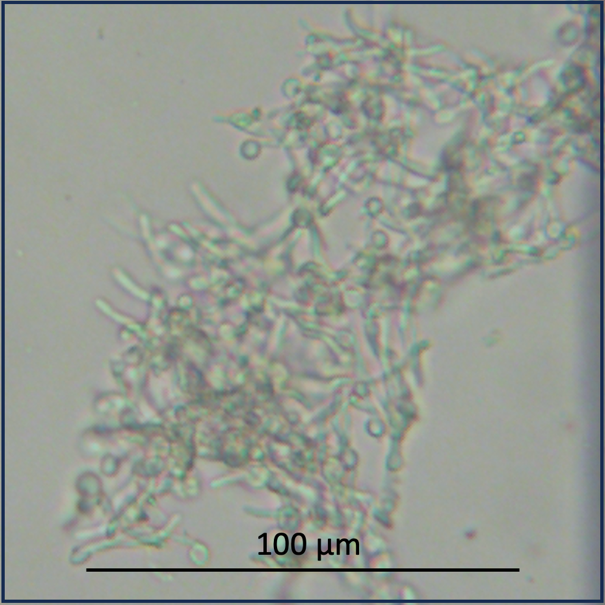


Conglomerate, Ascitic fluid

Legend: Morphology of *C. albicans* depending on the media, after 24 hours.

**Figure S2.** Heat production of *C. albicans* combined with different bacteria in different peritoneal fluids.


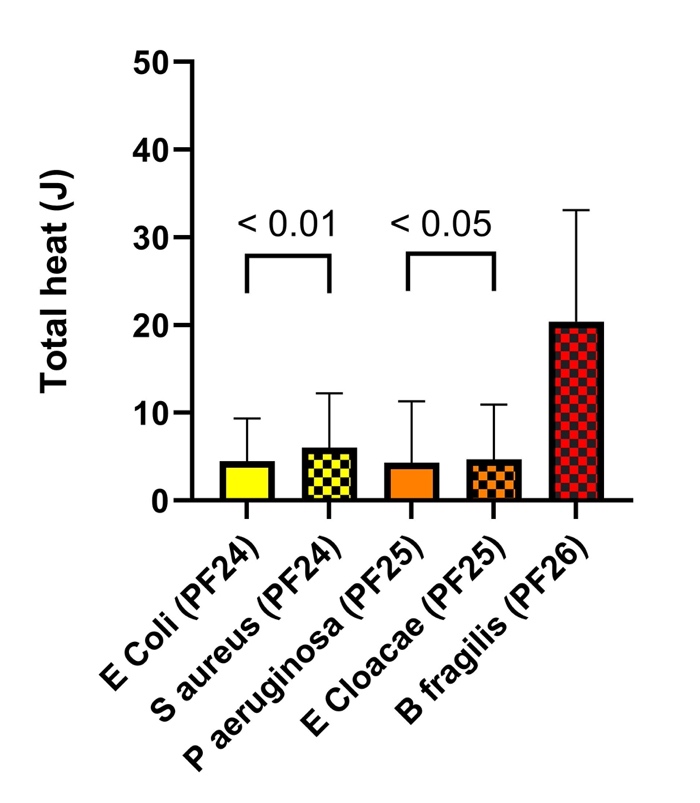


Legend: heat production is expressed as mean and standard deviation. Comparison between samples used ordinary one-way ANOVA.

Total heat production after 24 hours of *C. albicans* with different bacteria (*Escherichia* *Coli*, *Staphylococcus aureus*, *Pseudomonas aeruginosa*, *Enterobacter cloacae*, and *Bacteroides fragilis*), in peritoneal fluids 24 to 26. PF: peritoneal fluid.

**Figure S3.** Metabolic activity of *C. albicans* alone and with *S. aureus / E. coli* (**PF 24**) – *P. aeruginosa / E. cloacae* (**PF 25**) – *B. fragilis* (**PF 26**).


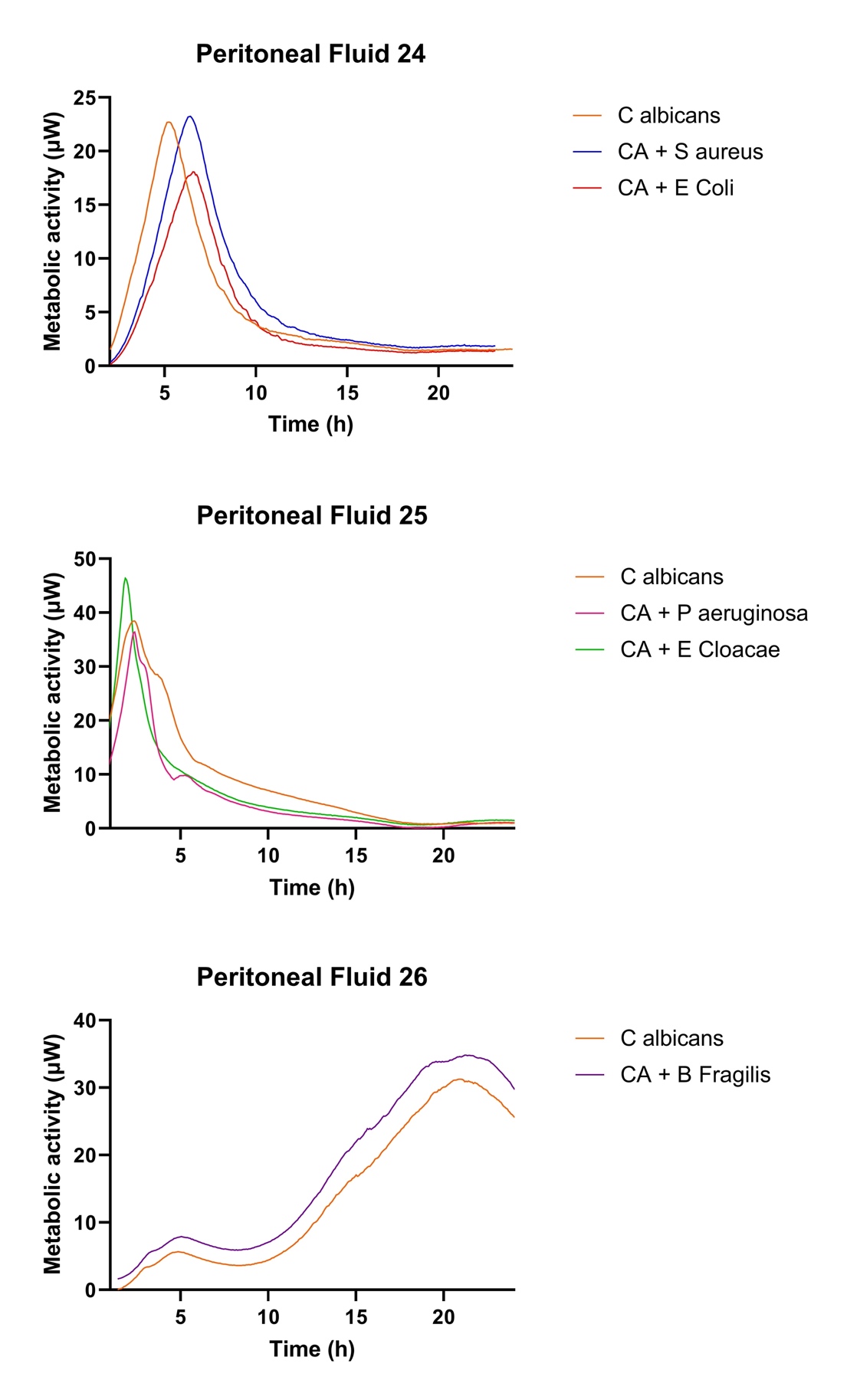


Interestingly, the metabolic activity of *C. albicans* with *S. aureus* or *E. Coli* (**PF 24**), and even more with *B. fragilis* (**PF 26**) persisted at 24 hours. Of note, this very high persistent activity in the **PF26** probably explains the highest metabolic rate reported **Figure 4D** (main manuscript). In the **PF 25** (combination with *P. aeruginosa* or *E. cloacae*), the metabolic activity disappeared at 24 hours.
